# Adipose-derived stem cells significantly increases collagen level and fiber maturity in patient-specific biological engineered blood vessels

**DOI:** 10.1371/journal.pone.0291766

**Published:** 2023-09-22

**Authors:** Bryan T. Wonski, Bijal Patel, Donna G. Tepper, Aamir Siddiqui, Loay S. Kabbani, Mai T. Lam

**Affiliations:** 1 Department of Biomedical Engineering, Wayne State University, Detroit, Michigan, United States of America; 2 Department of Plastic and Reconstructive Surgery, Henry Ford Health System, Detroit, Michigan, United States of America; 3 Department of Vascular Surgery, Henry Ford Health System, Detroit, Michigan, United States of America; National University of Singapore - Kent Ridge Campus: National University of Singapore, SINGAPORE

## Abstract

Tissue engineering has driven significant research in the strive to create a supply of tissues for patient treatment. Cell integration into engineered tissues maximizes functional capabilities, however, issues of rejection remain. Autologous cell sources able to solve this issue are difficult to identify for tissue engineering purposes. Here, we present the efficacy of patient-sourced cells derived from adipose (adipose-derived stem cells, ASCs) and skin tissue (dermal fibroblasts, PtFibs) to build a combined engineered tunica media and adventitia graft, respectively. Patient cells were integrated into our lab’s vascular tissue engineering technique of forming vascular rings that are stacked into a tubular structure to create the vascular graft. For the media layer, ASCs were successfully differentiated into the smooth muscle phenotype using angiotensin II followed by culture in smooth muscle growth factors, evidenced by significantly increased expression of αSMA and myosin light chain kinase. Engineered media vessels composed of differentiated ASCs (ASC-SMCs) exhibited an elastic modulus (45.2 ± 18.9 kPa) between that of vessels of undifferentiated ASCs (71.8 ± 35.3 kPa) and control human aortic smooth muscle cells (HASMCs; 18.7 ± 5.49 kPa) (p<0.5). Tensile strength of vessels composed of ASCs (41.3 ± 15.7 kPa) and ASC-SMCs (37.3 ± 17.0 kPa) were higher compared to vessels of HASMCs (28.4 ± 11.2 kPa). ASC-based tissues exhibited a significant increase in collagen content and fiber maturity- both factors contribute to tissue strength and stability. Furthermore, vessels gained stability and a more-uniform single-tubular shape with longer-term 1-month culture. This work demonstrates efficacy of ASCs and PtFibs to create patient-specific vessels.

## Introduction

For vascular repair, the gold standard autograft is in critically low supply [1, 2]. Synthetic grafts composed of polymers have shown a high incidence of thrombogenicity and cases of infection due to foreign body response and preference for bacteria to adhere to plastic surfaces [3, 4]. A completely biological-engineered vascular graft option would offer a superior solution. Fortunately, tissue engineering of vascular grafts has been a steadily developing field.

Current vascular tissue engineering methods have primarily involved producing tubular grafts with vascular cells from allogenic or xenogenic origins [5–7]. Though grafts using these cells types are useful for proof-of-concept, allogenic and xenogenic cells cannot be translated due to immunogenicity issues [8]. Grafts that do utilize allogeneic cells require decellularization to eliminate the immunogenic cell components [7, 9, 10], however, decellularization can cause issues of graft viability and eliminates the benefits of cellular proliferation; extracellular matrix (ECM) deposition; and cytokine production and crosstalk [11]. Following implantation, endogenous cells automatically populate the decellularized grafts, which takes time [10]. A meta-analysis of small-diameter tissue engineered grafts showed that longer recellularization rates significantly affected long-term patency and graft survival [12]. Hence, a cellularized graft would be ideal to maximize physiological functionality. Identifying a harvestable autologous cell source would solve such an issue. Specifically, an autologous source for smooth muscle cells (SMCs) and fibroblasts to build the tunica media and tunica adventitia, respectively, are needed.

First, in consideration of the tunica media, a direct autologous source of vascular SMCs is not a feasible source since sufficient sacrificial arteries from a patient are not readily available for SMC harvest, and the limited arterial segments possible to harvest would require extensive culture time to expand the few cells harvestable. Differentiating stem cells into vascular SMCs is a possible option. Adult stem cells harvestable from a patient could provide autologous cells. Beneficially, adipose-derived stem cells (ASCs) are abundant and easily harvested from patient adipose tissue obtained through either liposuction or lipectomy. In addition, ASCs derive from the same mesenchymal germ layer as SMCs, with prior evidence of successful differentiation towards an SMC phenotype [13]. ASCs have demonstrated the ability to express SMC contractile proteins *in vitro* following treatment with different growth factors [14–16]. Additionally, ASCs have been shown to improve the hemocompatibility of decellularized and synthetic polymer grafts, including demonstration of improved short-term patency and expression of smooth muscle contractile proteins in ASC-seeded poly(ester urethane)urea grafts [17–19]. Given the encouraging data on ASCs for SMC differentiation and use in an engineered graft, we explored the use of these patient-harvestable adult stem cells for application into our engineered vascular grafts. In addition, other groups have shown viability of ASCs for application into engineered grafts.

Secondly, to build the tunica adventitia, a harvestable source of fibroblasts is needed. The primary role of fibroblasts in the adventitia is to produce and deposit collagen to provide strength and structural integrity to ECM of the blood vessels [20]. Skin is a viable means to harvest autologous dermal fibroblasts [21]. Advantageously, fibroblasts are easily harvested from the patient skin, comprising a routine procedure in the clinic. Dermal fibroblasts have been shown to extensively promote collagen deposition in tissue-engineered vascular grafts [5, 6, 22], thus aiding in extracellular matrix development and tissue strength. Advantageously, while recent single-cell RNA sequencing analyses suggest some unique heterogeneity among fibroblasts from different anatomical regions, genes related to extracellular matrix organization and remodeling, such as collagen type 1 alpha 1 chain, remain highly conserved [23]. Therefore, dermal fibroblasts were identified as a viable, patient-harvestable option to mimic the microstructural ECM of the engineered adventitia. Furthermore, adventitial and dermal fibroblasts have demonstrated functional similarities in their responses to substrate stiffness through upregulating alpha smooth muscle actin and collagen types 1 and 3 alpha 1 chains [24, 25].

Here, we demonstrate generation of a patient-specific vascular graft. ASCs differentiated into SMCs and dermal fibroblasts were applied to our lab’s technique to engineer blood vessels. Our technique involves formation of rings of vascular tissue which are subsequently stacked into a tubular structure, creating the final vessel [26–28]. The advantage of our technique is that the cell type can easily be changed per needs of the application. ASCs differentiation towards the SMC phenotype was investigated using angiotensin II and a set of SMC growth factors typically found in growth media for SMCs [14, 29–31]. Harvested dermal fibroblast phenotype was verified for collagen I expression prior to application into the vascular rings. The differentiated SMCs (termed ASC-SMCs) and patient fibroblasts (termed PtFibs) were able to be successfully implemented in our engineered vascular graft technique. Patient-specific grafts were built from combined engineered media and adventitia vessels. The final tunica intima layer was not engineered into the patient-specific graft to take advantage of the natural process of endogenous endothelialization once implanted. Additionally, unpublished results on creating ring structures from endothelial cells showed that endothelial cells do not deposit sufficient ECM to create a stable ring structure.

## Materials and methods

### Cell isolation and culture

Human abdominal skin and adipose tissues were obtained with informed patient consent from ten healthy females (ages 24–62) undergoing elective abdominoplasty surgeries at Henry Ford Hospital—Main (Detroit, Michigan) and Henry Ford Medical Center–Cottage (Grosse Pointe Farms, Michigan) in accordance with Wayne State University (#054514M1E) and Henry Ford Health System (#Siddiqui_8958) Institutional Review Board (IRB) guidelines between February 2021-March 2022. All patient samples were deidentified prior to transfer from the hospital to Dr. Lam’s laboratory. Full thickness skin was collected from patients, and the dermis used for fibroblast isolation and hypodermis used for ASC harvest. Excised tissues were placed on ice and cells were extracted within 24h post-operation.

ASCs were extracted by mechanical digestion of adipose tissue followed by enzymatic digestion in 1 mg/mL collagenase type II. The cellular component was separated from the extract mixture by centrifugation at 1000 rpm. Following aspiration of the supernatant, the resultant pellet containing the stromal vascular fraction was resuspended in basic growth media consisting of 89% Dulbecco Modified Eagle Medium (DMEM), 10% fetal bovine serum (FBS), and 1% antibiotic-antimycotic. The stromal vascular fraction containing ASCs was expanded in culture plates. ASC cultures were used in the experiments between passages 3–6 to maintain ASC viability [32].

PtFibs were extracted using explant culture methods. Following ASC isolation, full thickness skin was cleared of remaining adipose tissue and cut into 3 x 3 mm sections. Skin sections were placed on 10% gelatin-coated culture dishes containing basic growth media with the dermis in contact with the gelatin surface. PtFibs were observed migrating from the excised skin sections onto the culture plate surface after 1–2 weeks. Skin sections were removed from the culture and extracted fibroblasts were expanded in 150 mm petri dishes and used for experiments at passages 4–9.

Human aortic smooth muscle cells (HASMC; PCS-100-012, ATCC, Manassas, VA) were used to generate the positive controls for the smooth muscle differentiation assays and tissue engineered tunica media. Smooth muscle growth media containing 88.5% DMEM; 5% of L-glutamine and fetal bovine serum; 1% antibiotic-antimycotic; 0.1% of recombinant human insulin (rH-insulin), recombinant human epidermal growth factor (rH-EGF), recombinant human fibroblast growth factor (rH-FGF) and ascorbic acid was used for HASMC expansion. Cells were utilized for experiments between passages 3–7.

### ASC differentiation

Angiotensin II was chosen as a demonstrated myogenic differentiation factor for ASCs, as it is known to upregulate smooth muscle contractile protein expression [14]. ASC passages 3–4 were seeded at a density of 2 x 10^3^ cells/cm^2^ in a differentiation media containing low glucose DMEM supplemented with 4% FBS, 1% antibiotic-antimycotic, and 2 μM angiotensin II (AngII). Two differentiation protocols were investigated: 1) ASC differentiation for 7 days in angiotensin II (group labeled “ASC-SMC I”); and 2) ASCs differentiated for 7 days in angiotensin II differentiation media, followed by culture for 7 days in smooth muscle growth media with smooth muscle growth factors of insulin, EGF, and FGF (group labeled “ASC-SMC II"). Media was changed in both groups every 2 days to maintain consistent differentiation factor exposure. Differentiated groups were analyzed for myogenic contractile marker expression using qRT-PCR analysis and immunofluorescence, and for functionally through a gel contraction assay.

### Preparation of engineered vascular tissue culture plates

Our engineered vascular constructs are fabricated using our previously established methods whereby vascular cell monolayers supported by a fibrin hydrogel are self-assembled into tissue rings which are then stacked to from the final vessels’ tubular structure [26–28, 33]. To fabricate the ring plates, 6-well culture dishes were surface coated with 2 mL of a 10:1 base to curing reagent mixture of poly(dimethylsiloxane) elastomer (PDMS). Once polymerized, a 6 mm diameter cylindrical PDMS post was adhered to the center of each well with additional PDMS.

To make the vessel plates, cylindrical poly(carbonate) tubing was attached to a poly(carbonate) base with acrylic glue. Poly(lactic) acid (PLA) removable posts with diameters of 6 mm and length of 15 mm were 3D-printed. Posts were filed to remove 3D printing burrs and surface coated with PDMS to protect the engineered vascular tissues. Post holders were attached to the center of the vessel dishes, and posts were fit into the holder. Both ring and vessel plates were sterilized first with a 1 h ethanol soak followed by 1 h of UV prior to use.

### Engineered vascular tissue fabrication–rings

**Fig 1:**
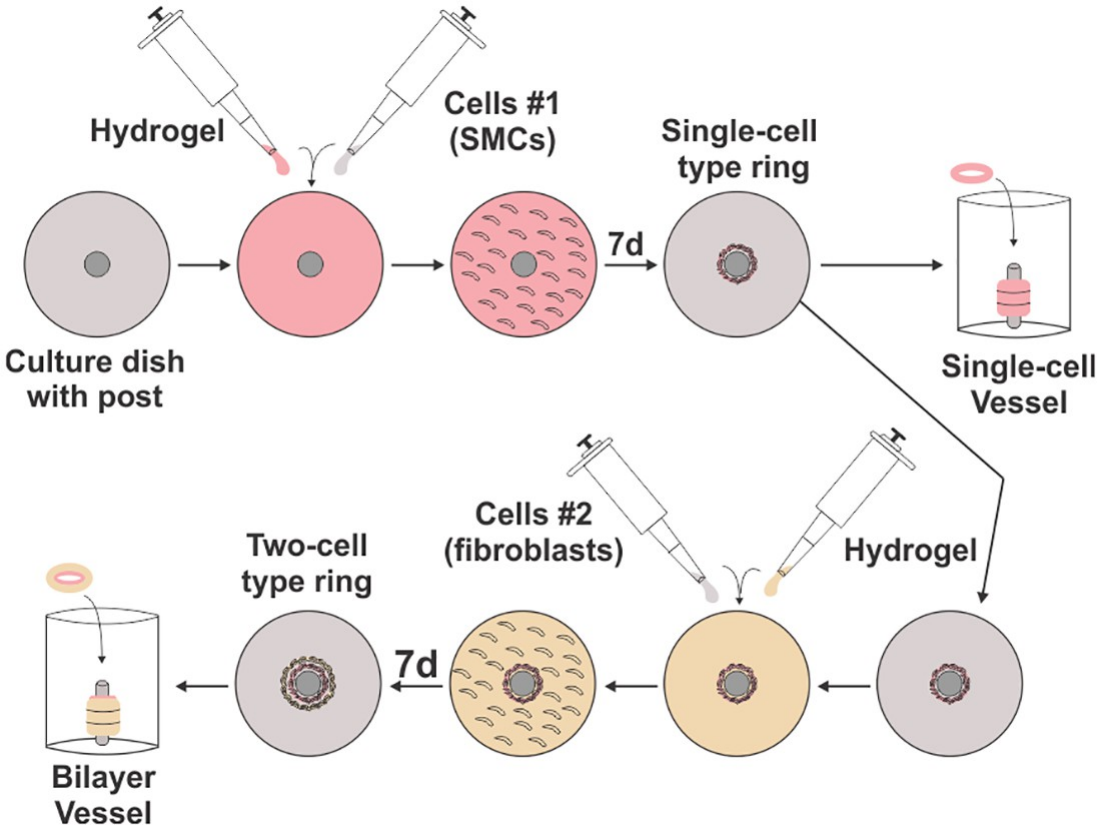
Diagram of engineered vessel fabrication protocol. The Ring Stacking Method (RSM) is outlined for single-cell and two-cell engineered rings and vessels. Monolayers of vascular cells are plated onto silicon elastomer-coated dishes with a central post. The bottom coating allows for the cell monolayer to detach from the bottom of the plate once formed. Aggregation of monolayer progresses towards the central post, creating a ring of vascular tissue. Single cell vessels are composed of either fibroblasts for the adventitia or smooth muscle cells for the media layer. Bilayer vessels combine the adventitia and media layers to form the complete graft.

Single-cell type (i.e. single layer) and two-cell type (i.e. bilayer) vascular tissue rings were created by seeding fibroblasts or fibroblasts and smooth muscle cells onto fibrin hydrogel in the ring plates with the central post for ring formation. Briefly, the rings form first by proliferation of the seeded cells into a cell monolayer. Attachment and detachment of the cell monolayer to the bottom of the plate is controlled by the silicone elastomer deposited on the bottom of the plate allowing for tissue self-organization, causing the monolayer to detach from the bottom once formed. Lastly, the monolayer aggregated towards the center and wraps around the post to form the vascular ring structure [26–28, 33]. The rings are then stacked into the final vessel form (Fig 1). Each cell type was individually tested by creating rings of ASCs (control), ASC-SMCs, HASMCs (control), or PtFibs. Cells prepared for seeding were suspended in 20 mg/mL bovine plasma fibrinogen for cells seeded inside the fibrin gels, or in ring culture media for seeding on top of the fibrin gels. Fibrin hydrogels were fabricated in the ring culture plates by depositing 0.5 mL of media composed of either SMC growth media for HASMC and ASC-based rings, or general growth media for PtFib rings. Next, 40 μL of 100 U/mL bovine plasma thrombin and 160 μL of fibrinogen containing 1 x 10^6^ cells were added to each well to create the fibrin hydrogel. Following fibrin gel polymerization, an additional 1 x 10^6^ cells suspended in their respective culture media, SMC growth media with 0.05 ng/mL transforming growth factor beta-1 (TGF-β_1_) for HASMC and ASC-based rings, or general growth media with 0.025 ng/mL TGF-β_1_ and 37.5 μL ascorbic acid for PtFib rings, were added dropwise on top of each gel. Plates were gently swirled to ensure uniform cell distribution followed by overnight culture in an incubator. After overnight incubation, media was changed followed by every 48 h thereafter for 7 days.

Combined tunica media and tunica adventitia “bilayer” rings were generated by forming adventitia rings around completed media rings. Media rings consisted of either ASC-SMC or HASMC cells depending on the group. For bilayer rings containing patient-derived cells, ASCs and PtFibs from the same patient (i.e. “patient-matched” cells) were used in the same bilayer rings to minimize effects from patient variability in the engineered tissues. Bilayer rings were constructed by first transferring fully formed ASC-SMC or HASMC rings on day 7 after initial cell seeding with sterilized forceps onto the PDMS posts of new ring plates into 0.5 mL of media of general growth media with PtFib cells for forming the adventitia ring. Rings were submersed in the PtFib cell media to coat the media rings which aided in the attachment between the engineered tunica media and adventitia. Next, PtFibs were seeded inside and on top of the secondary hydrogel. The PtFibs-hydrogel combination then aggregated towards the center of the plate to form an adventitia ring around the media ring, thus completing the bilayer ring.

### Engineered vascular tissue fabrication—Vessels

Single and bilayer vessels were created using our lab’s previously published Ring Stacking Method (RSM) in which engineered vascular rings are stacked and adhered together with fibrin glue to form a vascular tube. Briefly, following ring formation, depending on the group, 3 or 6 rings were transferred from their ring plates to a 6 mm diameter 3D printed PLA post within a custom vessel culture dish. Ring in the stacks were placed in contact with each other with sterile forceps to limit gaps and the stack coated with additional fibrin gel in 60 μL of 1:1 volumetric ratio of 100 U/mL thrombin and 20 mg/mL fibrinogen for every 3 rings stacked. Once the fibrin coating polymerized, vessels were cultured in their respective growth medias for 2 days prior to mechanical testing or for 4 weeks for long-term histological analysis.

### Quantitative Reverse Transcription Polymerase Chain Reaction (qRT-PCR)

Expression profiles of mesenchymal (endoglin, CD105; leukocyte common antigen, CD45; and thymocyte differentiation antigen-1, CD90) and smooth muscle (transgelin, TAGLN; smooth muscle alpha-2 actin, ACTA2; smoothelin, SMTN; and calponin-1, CNN1) gene markers were acquired via qRT-PCR. GAPDH served as the housekeeping gene. Total RNA was extracted from cell lysates of cultured HASMCs (n = 5 independent biological replicates) and ASC groups (n = 5 independent patient-matched biological replicates) using GeneJET RNA purification kits (FERK0731, ThermoFisher). Sample RNA concentrations were measured with a Qubit 2.0 Fluorometer and 1 μg of RNA from each sample was reverse transcribed with TaqMan Reverse Transcription Reagents kit (N8080234, Applied Biosystems Inc.). Quantitative PCR was performed on each of the patient-matched ASC groups and HASMC control biological samples in technical triplicates for PCR using 20 μL volumes consisting of PowerUp SYBR Green PCR master mix, PCR primers, and cDNA in a StepOne Plus Real-Time PCR system. Results were analyzed using the 2^-ΔΔCt^ method and presented as fold-change with respect to undifferentiated ASCs [34].

### Immunofluorescence imaging

Cellular production of smooth muscle contractile proteins alpha-smooth muscle actinin (αSMA) and myosin light chain kinase (MYLK) were analyzed in 2D cell cultures to evaluate differentiation protocols and in engineered vascular tissues to assess phenotype. For cell cultures, 30 x 10^3^ cells were seeded onto 0.1% gelatin coated glass slide covers and placed in PDMS coated culture wells. ASCs and HASMCs served as negative and positive controls for SMC differentiation, respectively. After 2 days of incubation, cells were fixed with 5% formalin. Cells were permeabilized with 0.1% Triton X-100 in TBS-Tween20 and blocked with TBS-Tween20 containing 3% bovine serum albumin. Mouse αSMA antibodies and Rabbit MYLK Polyclonal antibodies were diluted to concentrations of 4 μg/mL and 10 μg/mL, respectively, and used to incubate cells overnight at 4 °C in a wet box. The mouse αSMA monoclonal antibody developed by Little, C.D. was obtained from the Developmental Studies Hybridoma Bank, created by the NICHD of the NIH and maintained at The University of Iowa, Department of Biology, Iowa City, IA 52242. Coverslips were washed with PBS and incubated for 1 h with 10 μg/mL goat anti-mouse IgG (H+L) cross-adsorbed secondary antibody Alexa Fluor 488 and 15 μg/mL goat anti-rabbit IgG (H+L) cross-adsorbed secondary antibody Alexa Fluor 555 in PBS containing 1% bovine serum albumin. Finally, coverslips were washed with TBS-Tween20 and mounted with Fluoromount-G mounting medium containing DAPI.

Engineered tissues and the femoral artery control were fixed in 10% formalin followed by dehydration in alcohol and xylene. Dehydrated samples were mounted in paraffin and sectioned cross-sectionally at 7 μm thicknesses. Slides were cleared in xylene and rehydrated using a decreasing gradient of alcohol solutions and water. Prepared slides were immunofluorescent stained for αSMA antibody and DAPI.

Fluorescent images were acquired using an EVOS FL inverted fluorescent microscope and analyzed via ImageJ. Intensity quantification of 2D cultured cells was achieved by tracing non-overlapping individual cells (n = 70 per group from 3 independent samples) and measuring their area and integrated density at each fluorescent channel of interest. The intensities were normalized by the average of 3 background intensities for each image and presented as intensity per cell area. Images were batch edited for brightness to ensure consistency in minimal processing.

### Fibrin gel contraction assay

Cell contractility assays were conducted as a functional measure of smooth muscle differentiation. Total area of gels seeded with the SMC-differentiated cells were measured over time, with decreasing size of the gel correlating to higher cell contractility [15]. Fibrin hydrogels (n = 6 per group) were seeded with either ASC-SMCs, undifferentiated ASCs (control), or HASMCs (control). Hydrogel change in size was captured with time-lapse imaging over a 24 h period. Hydrogels were formed in 6-well plates by mixing 0.5 mL general cell growth media, 40 μL of 100 U/mL thrombin and 160 μL/mL of 20 mg/mL fibrinogen containing 500 x 10^3^ cells/mL. Following gel formation, 2 mL of smooth muscle growth media containing an additional 500 x 10^3^ cells/mL was carefully deposited dropwise on top of the fibrin hydrogel. Images of each gel were acquired immediately after seeding at 0 h and every hour for 6 h, and at 12, 21, and 24 h time points. Overall surface area of each gel was determined at each time point by outlining the gel perimeter in ImageJ followed by calculating the area in pixels and converting to cm^2^ by using a reference scale in each image.

### Mechanical analysis of engineered tissues

Mechanical properties of single and bilayer engineered vascular rings and vessels were assessed with circumferential tensile testing using a UStretch mechanical testing system (CellScale, Waterloo, Ontario, Canada) equipped with 5 N load cell. Tissue rings (n = 15–22 per group) were tested on day 7 of culture, while vessels (n = 5–6 per group) were tested 2 days post-stacking. Uniaxial circumferential tensile testing was performed at a strain rate of 0.4 mm/min until failure. Specimens were attached to the system by connecting metal hooks to the system clamps and inserting the hook through the engineered tissue lumen. Samples were placed at slack length and measurements for thickness and width were obtained using digital calibers at two points to determine cross-sectional area for stress calculations. Once stretched to failure, the elastic modulus (E), ultimate tensile strength (UTS), failure strength (FS), and percent elongation at failure were determined through stress-strain curve analysis.

### Tissue histology

The organization of cellular and extracellular matrix components of engineered vascular rings (n = 3 per group), long-term cultured vessels (n = 3 per group), and a fresh non-preserved cadaver femoral artery (control; n = 1) embedded in paraffin were analyzed histologically. Samples were exposed to 10% formalin for 24 h at room temperature followed by dehydration and temporary storage in 70% EtOH at 4 °C. Tissues were processed through a gradual 70 to 100% ethanol dehydration over 12 h followed by xylene. Next, samples were exposed to paraffin wax at 60 °C for 2 h, embedded into paraffin blocks, and sectioned at 7 μm thickness. Hematoxylin and eosin (H&E) staining was performed to determine overall cellularity; cellular alignment; the degree of extracellular matrix deposition; and organization of collagen. Collagen content in the samples was assessed using Masson’s trichrome (collagen marked blue) and Picrosirius red (collagen marked red) stains. Additionally, visualizing Picrosirius red stains under polarized light was used to differentiate thinner, immature collagen fibers (green to yellow) from dense, mature fibers (orange to red). Picrosirius red images (n = 4–5 per group) were further analyzed using ImageJ to quantify the percentage of collagen fibers per engineered tissue area. Verhoeff Van-Gieson (VVG) staining was performed to assess elastin fiber (black) content in tissue sections.

### Statistics

Immunofluorescence quantification, hydrogel contraction, vascular tissue mechanics, and collagen quantification data were presented as mean values ± standard deviation. PCR data was presented as the mean value with confidence intervals derived from the standard error [34]. Statistical significance for PCR gene expression was determined by independent-samples Kruskal-Wallis Tests with an alpha value of 0.05. A one-way ANOVA with a Tukey’s-b post-hoc test and an alpha value of 0.05 was used to determine statistical significance between groups in biochemical, mechanical, and collagen quantification datasets. Statistical significance for 1-month vessels collagen quantification was determined by an independent t-test with an alpha value of 0.05. All statistical analyses were calculated using SPSS.

## Results

### Angiotensin II with SMC growth factors induces smooth muscle-like phenotype in ASCs

**Fig 2:**
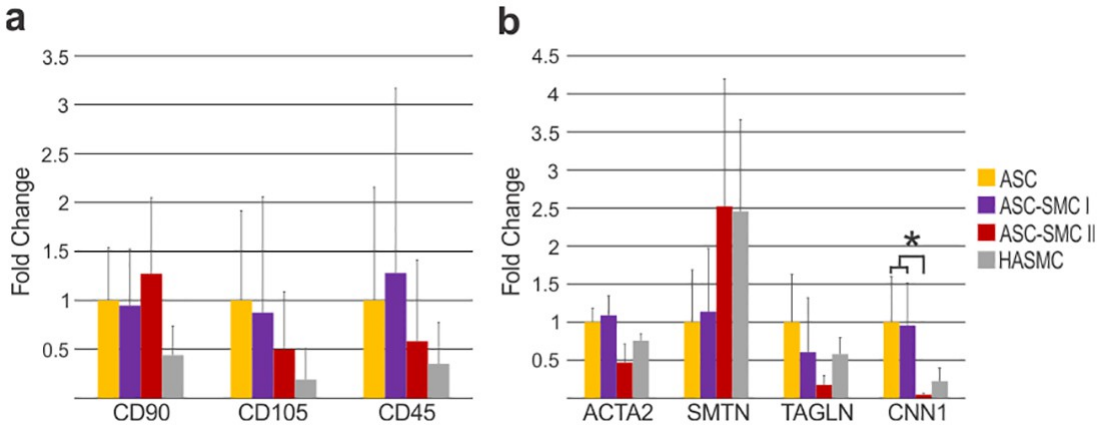
Relative mRNA expression profiles of the patient-derived cells. Characterization of patient cells relative to (**a**) ASCs using mesenchymal stem cell markers of CD90^+^_,_ CD105^+^, and CD45^-^; and (**b**) relative to smooth muscle markers smooth muscle alpha-2 actin (ACTA2), smoothelin, (SMTN), transgelin (TAGLN), and calponin-1 (CNN1). Mesenchymal markers positive markers of CD90 and CD105 were present in ASCs. Mesenchymal marker expression was lowest in HASMCs, indicating their more differentiated state comparatively. ASCs exhibited expression of muscle markers, likely due to their mesenchymal origins. Smoothelin smooth muscle contractile protein was highly expressed in ASC-SMC II and HASMC cells. * denotes significance between groups (p<0.05).

Previously, our lab demonstrated the ability to isolate CD105^+^/CD90^+^ mesenchymal stem cells from human adipose tissues [35]. Relative gene expression of CD105^+^, CD90^+^ and CD45^-^ were analyzed through qRT-PCR (Fig 2a). Average expression of CD105^+^ and CD90^+^ were slightly higher in ASC groups compared to HASMCs, though not significantly. CD45^-^ expression did not differ significantly between groups, however, the cycle threshold values obtained for CD45 were near the limit of detection, suggesting expression was limited in all groups examined. To determine the effect of growth factor supplementation on ASC smooth muscle-like differentiation, qRT-PCR for smooth muscle contractility proteins ACTA2, SMTN, TAGLN, and CNN1 was performed (Fig 2b). Interestingly, HASMC expression of ACTA2, TAGLN and CNN1 was found to be lower, but not significantly, compared to undifferentiated ASC and ASC-SMC I cells. Similarly, AngII + SMC-GM cultured ASCs had nonsignificant, lower expression of TAGLN and ACTA2, and significantly lower CNN1 expression relative to undifferentiated ASCs and ASC-SMC I (p<0.005). Yet, HASMCs and ASC-SMC II cells showed increased expression of SMTN compared to ASCs and AngII stimulated ASCs, though not significantly.

**Fig 3:**
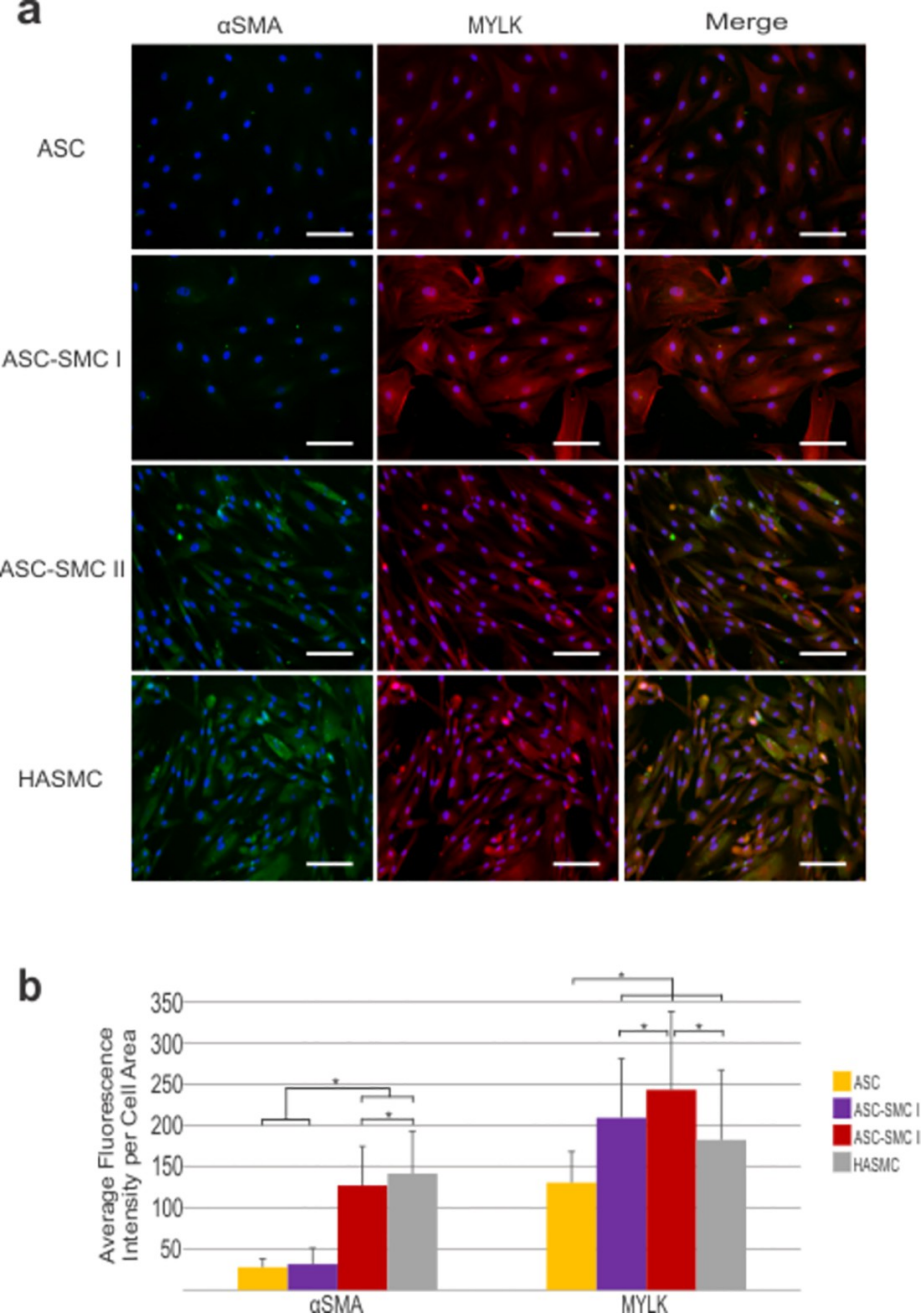
Smooth muscle differentiation of ASCs results in significant production of smooth muscle contractile proteins. (**a**) Alpha smooth muscle actin (αSMA) and myosin light chain kinase (MYLK) were detected via immunofluorescence in ASCs differentiated by angiotensin II (ASC-SMC I) or by angiotensin II followed by smooth muscle growth media (ASC-SMC II), and in human aortic smooth muscle cells (HASMCs) controls. Fluorescence images of cells stained for αSMA (green), MYLK (red) and DAPI (blue) were captured at 20x magnification (scale bar = 100 μm). (**b**) Quantification of average fluorescence signal intensity per cell area (n = 70) for each marker shows a significant increase in smooth muscle contractile protein expression following differentiation for the ASC-SMC II group. *denotes significance between groups (p<0.05).

The effect of culture conditions on ASCs contractile protein expression using HASMCs as a positive control was determined through immunofluorescence detection of αSMA and MYLK antibodies (Fig 3). Morphologically, ASCs cultured in presence of general growth media appear flat and elongated with multiple extensions resulting in a total cell area of 4549 ± 2160 μm^2^. While AngII cultured ASCs did not differ significantly in shape and size (5249 ± 2388 μm^2^), ASCs treated in AngII + SMC-GM and HASMCs were significantly smaller (p<0.05) with cell areas of 1438 ± 900.4 μm^2^ and 1198 ± 571.1 μm^2^, respectively. Average cell intensity of αSMA expression was increased significantly in ASC-SMC II cells compared to undifferentiated ASCs and ASC-SMC I cells indicating that the addition of the cytokines in the SMC GM significantly increased SMC differentiation over AngII alone. However, αSMA expression was significantly higher in HASMC controls compared to ASC-SMC II cells (p<0.05). MYLK protein expression in ASC-SMC I cells was higher relative to undifferentiated ASCs (p<0.05), producing intensities comparable to HASMCs. Additional expansion in SMC GM further increased MYLK production (p<0.05). Due to the increase in production of smooth muscle contractile proteins and transition towards a distinctly muscular fusiform morphology, the ASC-SMC II protocol was determined to be the optimal differentiation protocol and used in further experiments.

**Fig 4:**
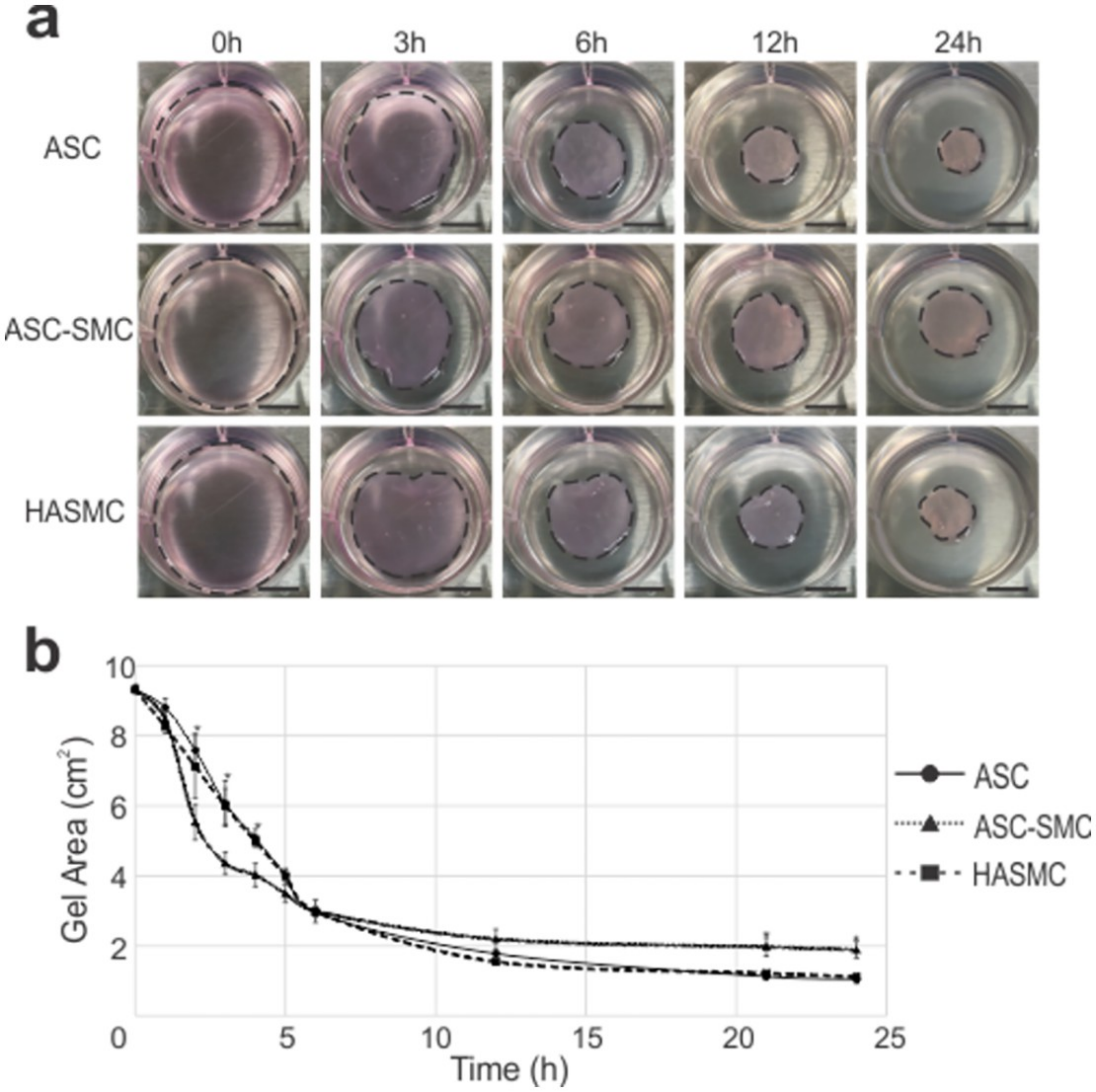
Functional assay results showed ASC-derived cells and HASMCs had similar contraction capacity. (**a**) Representative time-lapse images of hydrogels contracted by the cells over time. (**b**) Measurement of hydrogel area contraction by each cell type showed little difference in cell contraction capacity between ASCs, ASC-SMCs, and HASMCs suggesting ASCs are capable of contraction at a similar level to smooth muscle cells. * denotes significance between cell types at the designated time point. Scale bar = 10 mm.

Functionality of contractile proteins was assessed through cellular contraction of fibrin hydrogels. Representative images of cellularized hydrogels during sequential timepoints were collected (Fig 4). Initially, ASC-SMC II cells contracted gels significantly more than undifferentiated ASCs and HASMCs at 2–4 h time points (p<0.05). At 6 h, all gels had similar areas. At 21 h and 24 h time points, ASC-SMC II seeded gels were significantly larger (p<0.05) than ASC and HASMC samples.

### ASCs increase elasticity of engineered vascular tissues

**Fig 5:**
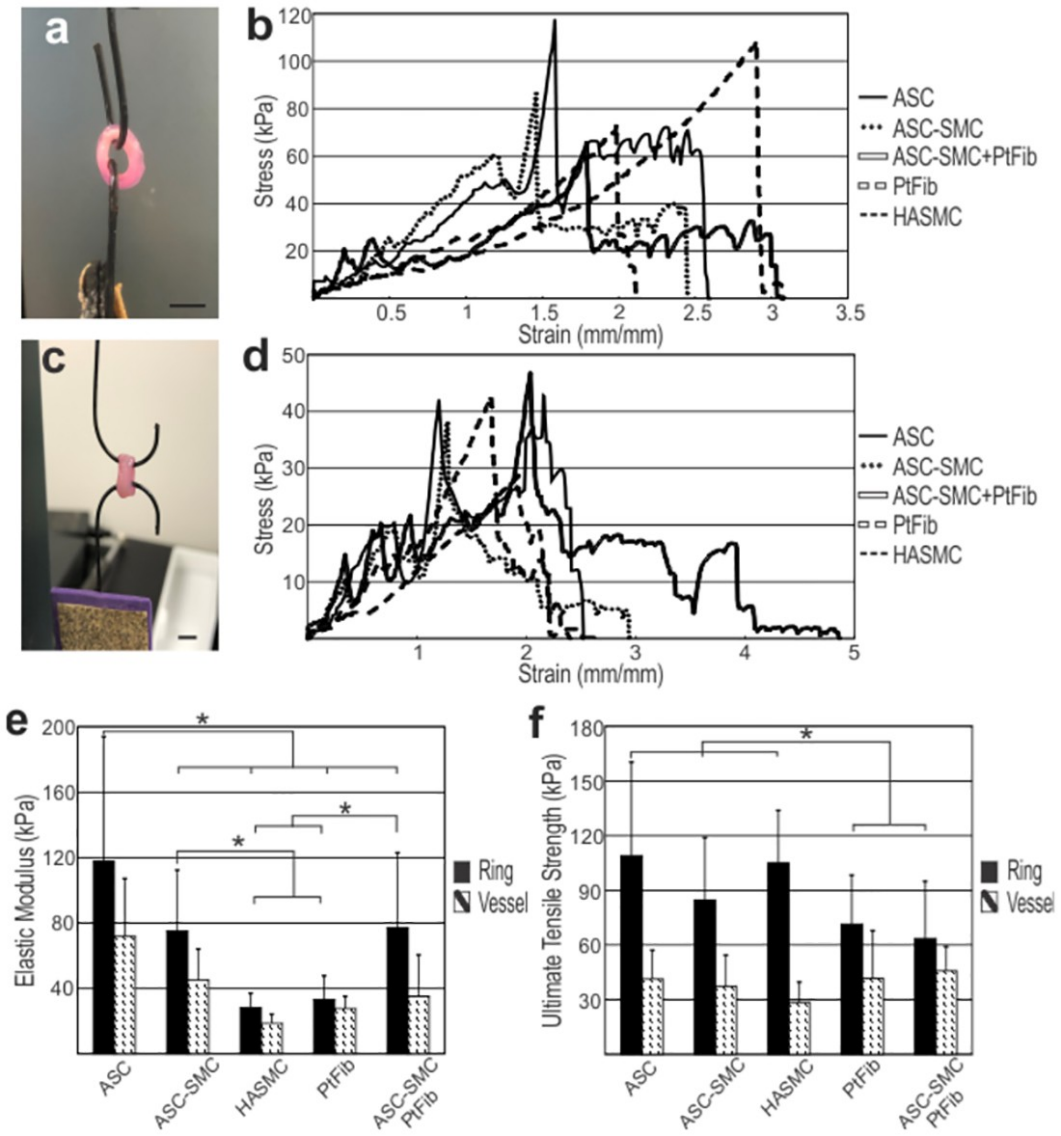
Ring and vessel tensile mechanics reveal increased elasticity in ASC-based engineered tissues. Tensile test setup for an (**a**) ASC-SMC + PtFib bilayer ring and (c) ASC-SMC + PtFib bilayer vessel. Stress-strain curves of (**b**) engineered ring mechanics and (**d**) showed increased (**e**) elastic modulus in ASC tissues relative to ASC-SMC and HASMC, while (**f**) ultimate tensile stress were similar. *denotes significance between groups (p<0.05). Scale bar = 6 mm.

Circumferential tensile mechanics of vascular rings varied significantly between cell types as shown by average stress-strain curves (Fig 5) and mechanical properties summarized in Table 1. ASC-SMC II differentiation resulted in a significantly lower elastic modulus of 75.0 ± 37.4 kPa compared to undifferentiated ASCs to 118 ± 75.9 kPa, indicating decreased stiffness following differentiation into the vascular smooth muscle phenotype. Both ASC-SMC II and undifferentiated ASC rings exhibited significantly higher elastic modulus than HASMC’s elastic modulus of 28.3 ± 8.69 kPa (p<0.05). However, the ultimate tensile strength of ASCs of 109 ± 51.4 kPa and ASC-SMCs of 84.6 ± 34.4 kPa rings did not differ significantly, and were comparable to that of HASMC rings of 105 ± 26.8 kPa. Furthermore, HASMC rings yielded a higher failure strength and percent elongation relative to ASC and ASC-SMC rings. Compared to other single layer rings, PtFibs had a significantly lower average ultimate tensile strength of 71.5 ± 26.8 kPa and an elastic modulus of 33.2 ± 14.5 kPa. Interestingly, the combination of ASC-SMC and PtFib layers to make bilayer rings significantly reduced ultimate tensile strength relative to ASC, ASC-SMC and HASMC tissues. The average maximum force of bilayer rings of 0.154 ± 0.065 N did not differ significantly from ASC, ASC-SMC, or PtFib single layer rings, which averaged 0.186 ± 0.068 N, 0.133 ± 0.069 N, and 0.134 ± 0.058 N, respectively. Moreover, bilayer rings averaged an elongation of 262 ± 51.4% before failure which was significantly higher than ASCs, ASC-SMCs, and PtFibs.

**Table 1:** Circumferential tensile properties of vascular tissue rings.

Ring Cell Type	E (kPa)	UTS (kPa)	FS (kPa)	Max F (N)	Elongation (%)
ASC	118 ± 75.9^b^^,^^c^^,^^d^^,^^e^	109 ± 51.4^d^^,f^	71.8 ± 32.7^c^^,^^e^	0.186 ± 0.068	212 ± 51.0^c^^,^^e^
ASC-SMC	75.0 ± 37.4^a^^,^^c^^,^^d^	84.6 ± 34.4^d^^,f^	57.0 ± 26.5^c^	0.133 ± 0.069^c^	213 ± 49.3^c^^,^^e^
HASMC	28.3 ± 8.69^a^^,^^b^^,^^e^	105 ± 26.8^d^^,f^	104 ± 26.3^a^^,^^b^^,^^d^^,^^e^	0.230 ± 0.063^b^^,^^d^^,^^e^	272 ± 29.6^a^^,^^b^^,^^d^
PtFib	33.2 ± 14.5^a^^,^^b^^,^^e^	71.5 ± 26.8^a^^,^^b^^,^^c^	66.6 ± 36.7^c^^,^^e^	0.134 ± 0.058^c^	219 ± 51.2^c^^,^^e^
Bilayer (ASC-SMC+PtFib)	77.0 ± 46.0^a^^,^^c^^,^^d^	63.5 ± 31.4^a^^,^^b^^,^^c^	42.8 ± 17.8^a^^,^^c^^,^^d^	0.154 ± 0.065^c^	262 ± 51.4^a^^,^^b^^,^^d^

^a^Statistically significant difference relative to ASC (p<0.05).

^b^Statistically significant difference relative to ASC-SMC (p<0.05).

^c^Statistically significant difference relative to HASMC (p<0.05).

^d^Statistically significant difference relative to PtFib (p<0.05).

^e^Statistically significant difference relative to Bilayer (p<0.05).

Vessel mechanics were assessed with 3-ring vessels. Mechanical analysis was performed 2 days following ring stacking (Fig 5; Table 2). Compared to individual rings, the ultimate tensile strength, failure strength, and elastic modulus of all vessels were comparatively lower due to a four-fold increase in cross-sectional area while average maximum force increased by two-fold. Additionally, due to individual rings rupturing at different stresses, two failure points, initial tearing at failure strength 1 (FS1) and complete breakage at failure strength 2 (FS2), were recorded as both provide information important to clinical applications. Surprisingly, ultimate tensile strength, maximum force, and both failure strengths did not differ statistically between the groups, suggesting similarity in mechanical properties of the HASMC and ASC-derived engineered vessels. The average ultimate tensile, elastic modulus, primary and secondary failure strength of undifferentiated ASC vessels were 41.3 ± 15.7 kPa, 71.8 ± 35.3 kPa, 38.7 ± 18.1 kPa, and 18.2 ± 6.22 kPa, respectively. ASC-SMC vessels had average ultimate tensile strength, elastic modulus, primary and secondary failure strengths of 37.3 ± 17.0 kPa, 45.2 ± 18.9 kPa, 34.5 ± 19.5 kPa, and 6.02 ± 1.91 kPa, respectively. Elastic moduli of vessels made of PtFibs, HASMCs, and bilayers consisting of ASC-SMCs and PtFibs were significantly lower relative to ASCs (p<0.05) with average values of 27.7 ± 7.49 kPa, 18.7 ± 5.49 kPa, and 35.2 ± 25.3 kPa. Furthermore, bilayer vessels had significantly longer elongation before secondary failure, relative to ASC, HASMC, and PtFib vessels, with an average of 311 ± 57.0%. Overall, culturing ASCs in differentiation medias prior to ring and vessel fabrication primarily effected elastic modulus of tissue cultures while maintaining average ultimate tensile strength. Formation of a tunica media-adventitia bilayer did not significantly improve ultimate tensile strength, however, bilayer rings and vessels improved elongation before failure relative to PtFib groups and ASC-SMC rings.

**Table 2:** Circumferential tensile properties of tissue engineered vessels (3-rings per vessel).

Vessel Cell Type	E (kPa)	UTS (kPa)	FS1 (kPa)	FS2 (kPa)	Max F (N)	Elongation (%)
ASC	71.8 ± 35.3^b^^,^^c^^,^^d^	41.3 ± 15.7	38.7 ± 18.1	18.2 ± 6.22	0.359 ± 0.165	212 ± 35.3
ASC-SMC	45.2 ± 18.9	37.3 ± 17.0	34.5 ± 19.5	6.02 ± 1.91	0.341 ± 0.117	285 ± 55.0
HASMC	18.7 ± 5.49^a^	28.4 ± 11.2	28.2 ± 11.4	15.1 ± 12.7	0.295 ± 0.079	230 ± 34.3
PtFib	27.7 ± 7.49^a^	41.7 ± 26.1	40.7 ± 26.4	11.8 ± 6.32	0.267 ± 0.175	210 ± 49.2
Bilayer (ASC-SMC+PtFib)	35.2 ± 25.3^a^	45.7 ± 13.3	45.6 ± 13.3	10.1 ± 7.45	0.272 ± 0.103	311 ± 57.0^a^^,^^b^^,c^

^a^Statistically significant difference relative to ASC (p<0.05).

^b^Statistically significant difference relative to HASMC (p<0.05).

^c^Statistically significant difference relative to PtFib (p<0.05).

^d^Statistically significant difference relative to Bilayer (p<0.05).

### ASCs significantly increase collagen content in engineered vascular tissues

**Fig 6:**
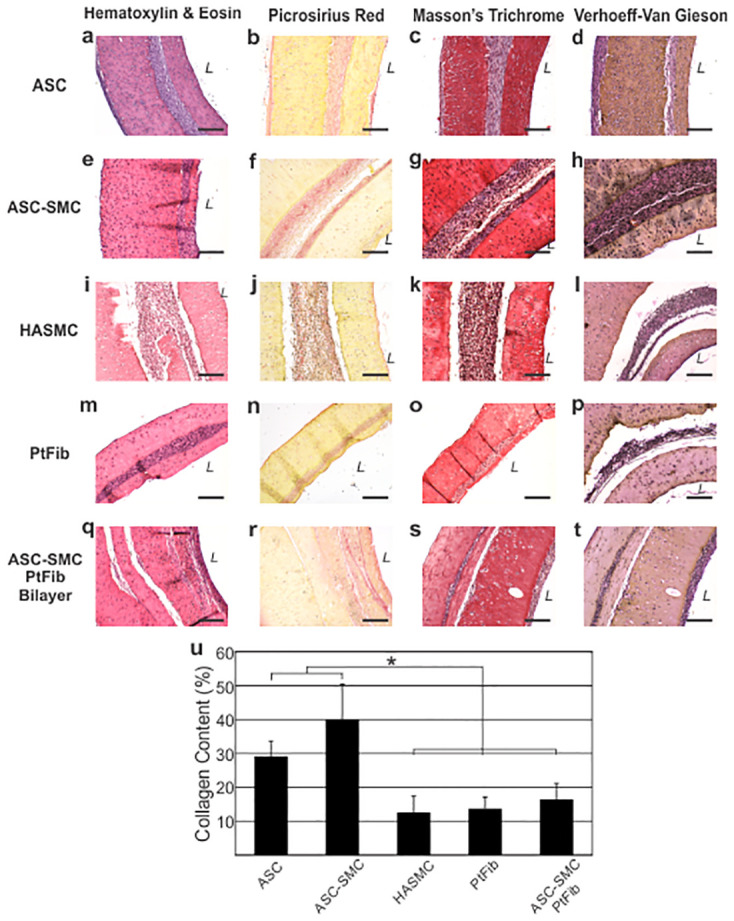
Patient cell-based engineered tissues exhibit organized cellularity and extracellular matrix. Histological analysis of tissue rings composed of (**a-d**) ASC, (**e-h**) ASC-SMC, (**i-l**) HASMC, (**m-p**) PtFib, and (**q-t**) ASC-SMC + PtFib bilayer. Tissue organization as revealed in H&E, Picrosirius Red, and Masson’s Trichrome showed circumferential organization in all engineered tissues. (**u**) Quantitative image analysis of Picrosirius Red illustrates increased collagen content in ASC-derived tissues as a percentage of tissue area. Verhoeff-Van Gieson stain showed minimal to no evidence of elastin fibers in the engineered tissues. *L* denotes lumen. Scale bar = 200 μm.* denotes statistical significance between connected groups (p < 0.05).

Cellular organization, extracellular matrix content, and contractile protein expression were identified through staining of tissue engineered rings (Fig 6). After 7 days in culture, cell monolayers in all cell groups aggregated into self-assembled rings with a provisional fibrin gel coating to aid in stabilizing the monolayer as it aggregated into rings. The cells in the rings aligned circumferentially around the central post in the plate. Ring thickness varied slightly.

Collagen was noticeably abundant in the ASC and ASC-SMC rings, while HASMC and PtFib rings appeared to have less collagen deposition. Image analysis quantification of the percentage of collagen content per total ring area confirmed these observations. Collagen content in HASMC, PtFib, and ASC-SMC bilayer rings made up 12.6 ± 4.79%, 13.7 ± 3.47%, and 16.4 ± 4.69% of total ring area, which was significantly less than both ASC based groups (Fig 6u; p<0.05). ASC-SMC rings contained significantly more collagen per ring area with an average of 39.9 ± 10.4% relative to the 29.2 ± 4.54% observed in ASC rings. Under polarized light, collagen fibers in ASC rings displayed green whereas collagen in ASC-SMC rings appeared primarily green and yellow signifying more maturity. Collagen fibers were nearly undetectable in PtFib and HASMC samples. In ASC-SMC bilayer vessels, more mature collagen fibers, indicated by yellow and red, were present in the ASC-SMC ring. Expectedly, Verhoeff-Van Gieson stains revealed absence of elastic fibrils within all groups (Fig 6).

**Fig 7:**
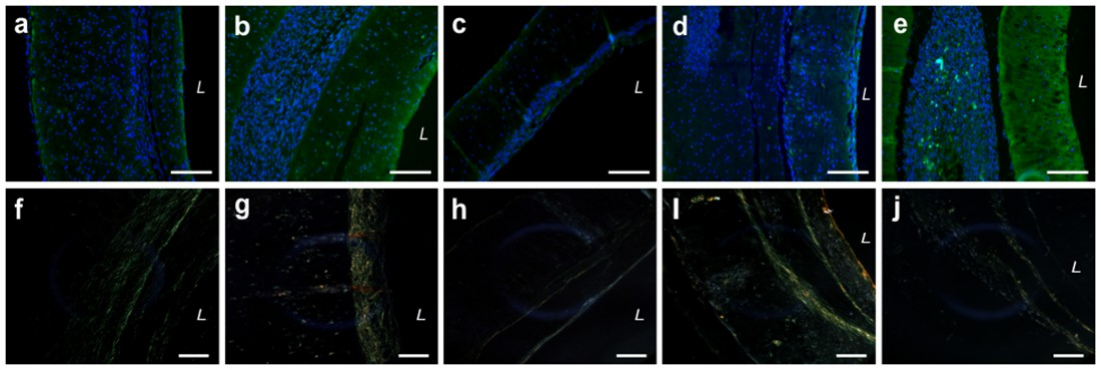
ASC-based engineered tissues show marked αSMA expression and mature collagen. (**a-e**) Immunofluorescence analysis of smooth muscle contractile protein αSMA (green) levels shows comparable expression in ASC-SMC tissues relative to control HASMC tissues. (**f-j**) Polarized light analysis of rings show primarily green immature collagen fibers across the engineered tissues, with some evidence of more mature yellow to orange fibers in the ASC-SMC tissues. (**a,f**) ASC, (**b,g**) ASC-SMC, (**c,h**) PtFib, (**d,i**) ASC-SMC + PtFib Bilayer, and (**e,j**) HASMC. Scale bar = 200 μm.

The effects of tissue fabrication on cellular production of αSMA was determined through immunofluorescence imaging (Fig 7). PtFib and HASMC rings were used as negative and positive controls, respectively. Despite limited production seen in 2D cultures, ASC rings stained positive though with limited expression. The limited stain present in the primary cellular layer of ASC and PtFib rings was expected as methods for ring formation requires minute concentrations of TGF-β_1_, a known stimulator of αSMA [36]. ASC-SMC tissues were also stained positive. Notably, a distinct difference in αSMA intensity can be seen between ASC-SMC and PtFib layers within the bilayer ring.

### Long-term culture bilayer vessel histological analysis

**Fig 8:**
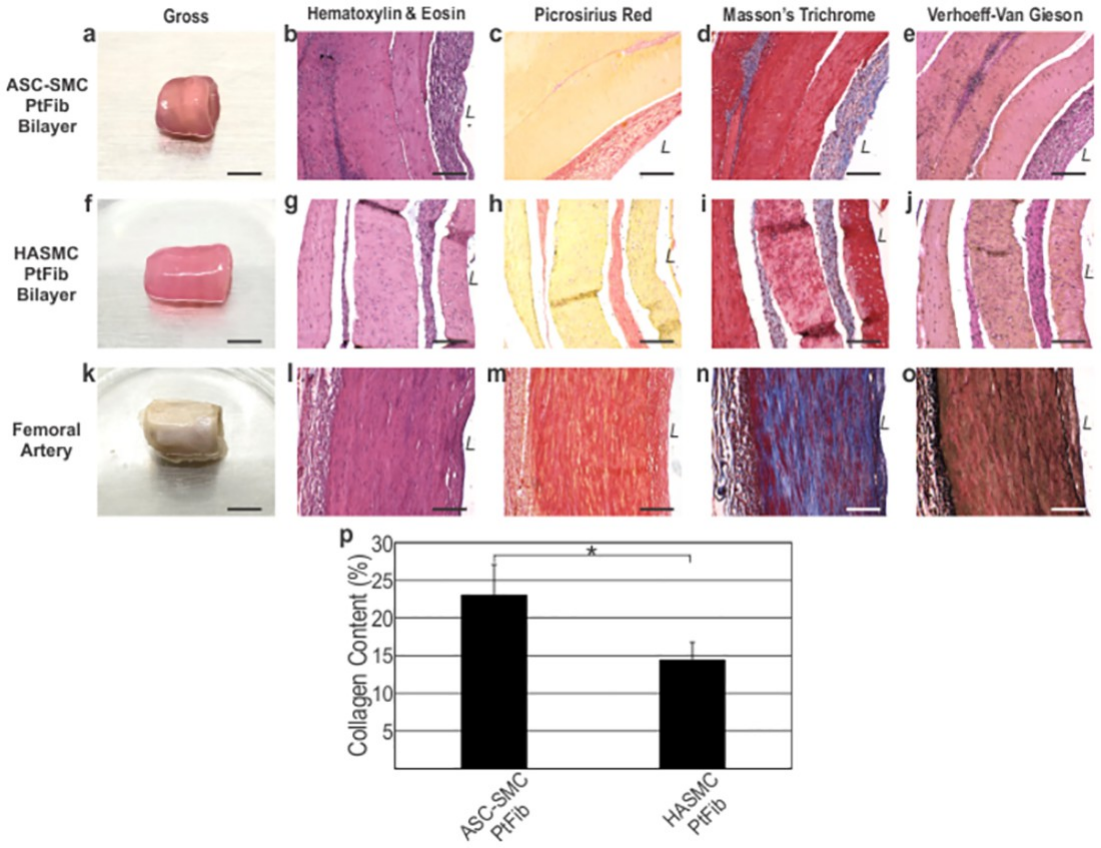
ASC-based engineered vascular tissues maintain cellular organization and content over time. (**a, f, k**) Gross and histology following 1-month in culture showed (**a-e**) ASC-SMC bilayers and bilayers constructed of (**f-j**) HASMCs retain circumferential alignment and maintain two distinct cellular layers when cocultured with PtFibs. ASC-SMC bilayers exhibit significant hydrogel degradation on the luminal side of the vessel. (**k-o**) Human cadaver femoral artery was used as a native vessel control. (**p**) Collagen quantification of Picrosirius Red shows significantly more collagen content in ASC-SMC tissues. *L* denotes lumen. Scale bar for gross images = 5 mm. Scalebar for histological images = 200 μm. * denotes statistical significance (p < 0.05).

**Fig 9:**
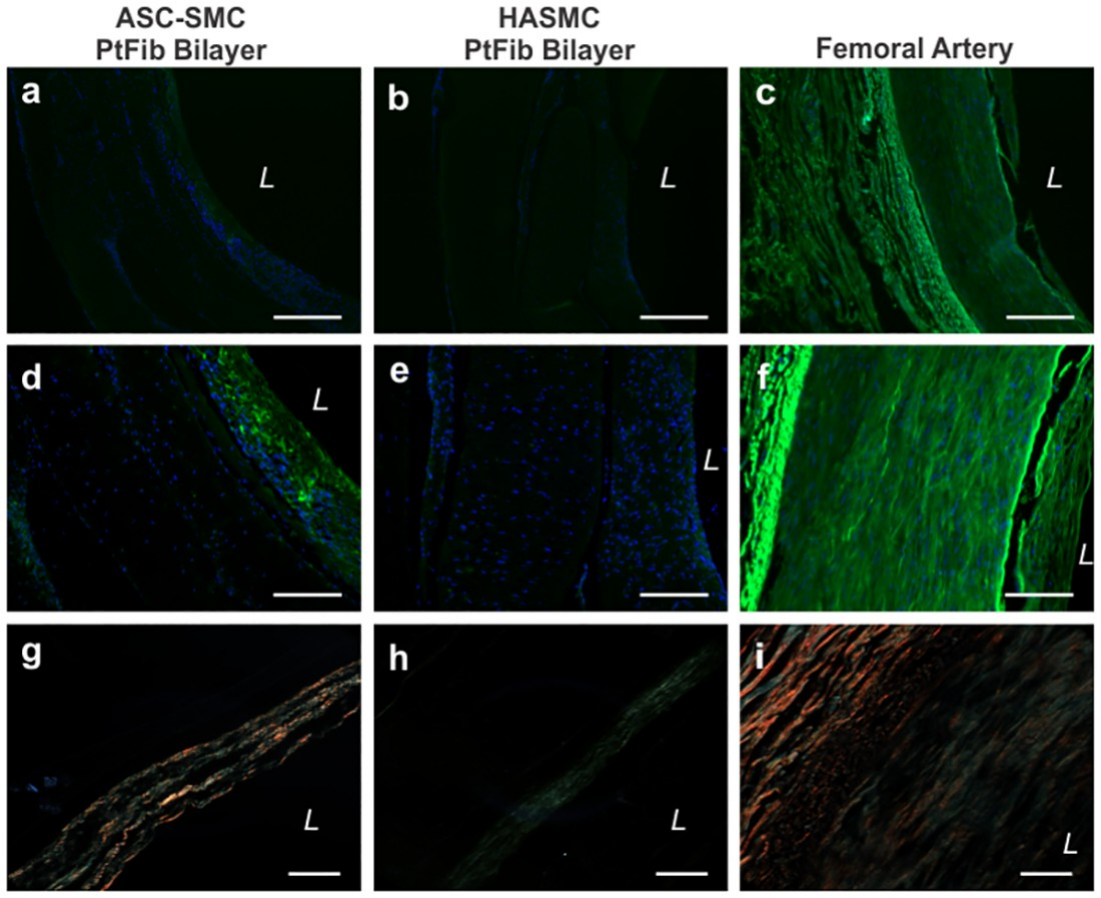
ASC-based patient-specific engineered bilayer vessels show higher αSMA expression and mature collagen compared to HASMC vessels. αSMA (green) immunofluorescence stain showed higher intensity in the ASC-SMC bilayer compared to HASMC bilayers. Polarized light showed more mature yellow, orange, and red fibers in the ASC-based vessels, similar to the femoral artery, whereas the HASMC vessel was primarily composed of green immature collagen fibers. *L* denotes lumen. Scalebar = 200 μm.

Vessel long-term viability and matrix remodeling were assessed in bilayer vessels comprised of a PtFib tunica adventitia and either ASC-SMC or HASMC derived tunica media (Fig 8). Histologically, engineered tissues preserved circumferential alignment in both distinct layers as shown by H&E stains. As desired, ASC-SMC bilayers degraded the provisional luminal side fibrin gel and the cellular layers proliferated, creating a more robust tubular structure indicative of a native vessel. Sections of a fresh cadaver femoral artery were used as the reference for native artery organization and extracellular matrix content. Prolonged static culture resulted in significant deposition of collagen isolated to the cellular areas, as shown by the red and blue stained fibers. Quantification of the picrosirius stained tissues determined the collagen content per area in ASC-SMC bilayers was an average of 23.1 ± 3.98%, which was significantly higher (Fig 8p; p<0.05) than the 14.5 ± 2.26% present in HASMC bilayers. Additionally, polarized light showed the collagen fibers present in ASC-SMC bilayers were more mature compared to those in HASMC bilayers (Fig 9a–9c). However, cadaver femoral artery samples exposed to polarized light indicated that the smooth muscle layer of native arteries contains primarily immature green fibers while the adventitia contains mature orange-red collagen. Elastin deposition was not present in either engineered bilayer while large elastin fibrils are seen in the native femoral artery.

Retainment of contractile smooth muscle phenotype following long-term static culture was determined through immunofluorescence detection of αSMA in ASC-SMC and HASMC using the femoral artery as a positive control (Fig 9d–9i). Both bilayer groups demonstrated positive stains near the lumen within the tunica media and less intensity with the PtFib derived tunica adventitia. Interestingly, αSMA intensity was more prominent in the ASC-SMC bilayer relative to the HASMC bilayer.

## Discussion

This work demonstrates the feasibility of utilizing patient dermal fibroblasts and adipose-derived stem cells for creating a patient-specific tissue engineered vascular graft. The key factors addressed here were determining a harvestable, abundant source of autologous cells; identifying a viable vascular differentiation protocol to produce vascular smooth muscle cells; and establishing a vascular tissue engineering method conducive to patient cell application.

Viable autologous cell sources have been explored for decades. Many studies have investigated the utility of various stem cell sources, though the field has yet to solidify a workable solution. We identified tissues easily harvestable by investigating tissues commonly extracted during cosmetic surgeries, namely skin and adipose tissue. Dermal fibroblasts are easily harvested from a small skin sample from a patient. Adipose tissue is commonly extracted from patients through liposuction procedures. In our work, patient dermal fibroblasts and ASCs were matched to the same patient for each vessel, thus creating an actual patient-specific vessel. Endothelial cells (ECs) for building the intima layer were not explored since there is no patient source of harvestable ECs. Additionally, we did explore differentiation of ASCs towards the EC phenotype, however differentiation from ASCs to ECs did not result in detectable expression of EC markers (unpublished data).

The key to determining the usefulness of these cells lays in their feasibility for vascular application. ASCs were not explored for use in the adventitia, as the primary cell type in the adventitia is the fibroblast, hence dermal fibroblasts were deemed more relevant and closer to the desired phenotype. Fibroblasts from different anatomical tissues do vary somewhat in phenotype, however, their primary function of collagen production is mostly conserved allowing for fibroblasts from other tissues to be applied to the collagen-rich tunica adventitia. Advantageously, ASCs are easily harvestable adult stem cells. The next step was then to characterize their differentiation potential into vascular smooth muscle cells to fabricate the engineered tunica media. The final layer, the tunica intima, is composed of endothelial cells (ECs). There is currently no source of harvestable autologous adult ECs. In research studies, human umbilical vein endothelial cells (HUVECs) are the standard, however these cells have a very different phenotype from vascular ECs. Fortunately, the human body naturally endothelializes any tubular-shaped structures placed into the circulatory system [37, 38]. This naturally occurring phenomenon can be employed to optimally create an intimal layer in the engineered bilayer vessels composed of a media and adventitia, hence the intima will potentially be automatically established once the graft is implanted. Therefore, the intima was not focused on during development of this patient-specific vascular graft.

Previous studies have shown angiotensin II facilitates the production of smooth muscle-related contractile protein in ASCs [14, 39]. In the present work, ASCs cultured in AngII for 7 d did not significantly alter αSMA gene or protein expression, however, myosin light chain kinase (MYLK) expression was increased as determined by immunofluorescence antibody detection (Fig 3). MYLK is an enzyme responsible for phosphorylation of myosin II during muscle contraction thus represents functional capacity. An adaptation to this differentiation protocol was established to promote cell proliferation following AngII stimulation by culturing cells in smooth muscle growth media following AngII differentiation. This process drastically increased αSMA and MYLK antibody signal intensity and prompted cell morphology progression towards the fusiform shape present in HASMCs. Interestingly, despite the increase in protein expression detected through immunofluorescence, gene expression of all smooth muscle related proteins except smoothelin was found to be higher in undifferentiated ASC relative to AngII + SMC-GM treated ASCs and HASMCs controls (Fig 2). Additionally, functional contractility in undifferentiated ASCs was nearly identical to HASMCs in fibrin gel contraction assays, while AngII + SMC-GM treated ASCs initially contracted gels significantly more followed by limited change after 12h (Fig 4). Overall, our results correlate with others studying ASC differentiation towards the smooth muscle lineage in that contractile protein expression can be increased through biochemical factors, however, variability between donor tissues will need to be considered [15, 39].

The use of undifferentiated ASCs and pre-differentiated ASCs treated with the AngII + SMC-GM protocol (ASC-SMC IIs) as a smooth muscle source for tissue engineering was further assessed by tissue mechanical and histological properties. The primary difference between both ASC-based modular tissue rings and HASMC-derived rings was the elastic modulus (Fig 5; Table 1). ASC-SMC rings had significantly lower average elastic modulus than ASC tissues, though HASMCs were significantly lower than both groups. Histological analyses showed the increased rigidity of ASC-based rings was primarily due to the significant deposition of collagen produced by both ASC groups (Fig 6). ASC-SMC II differentiation resulted in a significantly lower elastic modulus of 75.0 ± 37.4 kPa compared to undifferentiated ASCs to 118 ± 75.9 kPa, meaning that ASCs differentiated into SMCs exhibited lower stiffness, similar to smooth muscle phenotype. However, differentiated ASC rings exhibited a much higher elastic modulus compared to HASMC rings, denoting that the differentiated ASCs’ phenotype lays mechanically in between undifferentiated ASCs and HASMCs. This “in-between” elastic phenotype may be advantageous in a vascular milieu where tissue compliance is key to proper vascular function. The αSMA expression of differentiated ASCs was in between undifferentiated ASCs and HASMCs, further indicating their phenotype. Fortunately, the ultimate tensile strength, maximum force, and both failure strengths did not differ statistically between the groups, indicating that the strength exhibited by tissues composed of differentiated ASCs was similar to that of HASMCs.

In addition to assessing the use of patient cells, this work adapted our lab’s previous modular tissue engineering techniques to create tunica media-adventitia bilayers. Bilayers were successfully constructed. ASC-SMC and HASMC rings and vessels showed positive immunofluorescence staining for αSMA. Unexpectedly, the addition of a fibroblast adventitia exterior ring significantly reduced the ultimate tensile strength of the ASC-SMC + PtFib bilayer rings relative to ASC-SMC rings, although this effect was not present in vessels. This may be explained by the significant difference in average cross-sectional area between ASC-SMC (1.61 ± 0.472 mm^2^) and bilayer tissue rings (2.48 ± 0.471 mm^2^), which was negligible in vessels, since the average maximum force between both groups in ring and vessel form were not significantly different. The major contribution of strength in bilayer tissues was attributed to the ASC-SMC layer as PtFib rings and vessels had the lowest average maximum force and exhibited limited collagen production. In comparison, the mechanics of our engineered vascular tissues were significantly lower than those of human saphenous vein previously reported by our group, which had an average ultimate tensile strength and elastic moduli of 1060 ± 155 kPa and 2980 ± 409 kPa, respectively [26]. However, the tissue engineered vessels in this work were analyzed immediately after fabrication and we have recently shown improvement of biologically-engineered vessel mechanics over time in culture [40]. Additionally, we have adopted a new fibroblast embedded coating method capable of withstanding an average burst pressure of 229 ± 23.8 mmHg following 16-weeks of culture [40]. The inherently weak mechanics of biologically-engineered vessels requires prolonged culture periods, which combined with the time needed to proliferate cells for fabrication, currently limits clinical translatability for immediate procedures. A potential solution that we aim to incorporate is mechanical conditioning to increase in vitro maturation rates, and consequently, mechanical rigidity [6, 41].

One-month culture of bilayer vessels made of a PtFib-derived tunica adventitia and either ASC-SMC- or HASMC-derived tunica media showed significant deposition of collagen within the cellular regions (Figs 8 and 9). Compared to ASC-SMC bilayer rings cultured for 1 d, longer term culture ASC-SMC bilayers preferentially degraded the luminal side fibrin gel and cellular layers proliferated, increasing the cellular content. The engineered media and adventitia remained separate after long-term culture as two distinct cellular regions as seen in all samples. Additionally, elastin deposition was not present in any tissues in this study. In vitro elastogenesis has been challenging throughout the field of tissue engineering. Studies have shown elastin production requires prolonged culture under mechanical stresses such as pulsatile perfusion or cyclic stretch [41].

In terms of clinical application, adipose tissue is feasibly harvested from patients using liposuction procedures. Lipoaspirated adipose tissue has viably isolated healthy ASCs [42, 43]. Dermal fibroblasts are regularly harvested in the clinic with skin samples [44]. However, variability in ASC differentiation capacity based on age and pre-existing health conditions, such as diabetes, has been previously reported [15, 39]. Further studies assessing the differentiation capacity of ASCs from individuals requiring grafts for vascular bypass or hemodialysis vascular access are warranted. Nevertheless, ASCs may be a promising alternative, minimally-invasive cell source of smooth muscle-like cells for younger populations in need of a vascular graft capable of growing and remodeling throughout development. While the present work was limited to female tissues, no noticeable trends between donor age and ASC differentiation or tissue mechanics were observed despite variation in gene expression and mechanical properties between patient samples. Hence, autologous cell harvest is possible for the cell types tested in this work, thus demonstrating the efficacy of producing patient-specific vascular grafts.

## Conclusion

This study demonstrated the efficacy of ASCs and dermal fibroblasts as viable autologous cells for generating patient-specific vascular grafts. ASCs were successfully differentiated into vascular smooth muscle cells to engineer the tunica media. Patient fibroblasts were applicable to engineering the tunica adventitia. Advantageously, ASCs significantly increased collagen content and maturity in the engineered vessels. Combined, the engineered media and adventitia formed a complete patient-specific vessel characterized by increased elasticity for compliance needed for blood flow. Ongoing work entails in vivo implantation studies.
